# The value of GRASP on DCE-MRI for assessing response to neoadjuvant chemotherapy in patients with esophageal cancer

**DOI:** 10.1186/s12885-019-6247-3

**Published:** 2019-10-24

**Authors:** Yanan Lu, Ling Ma, Jianjun Qin, Zhaoqi Wang, Jia Guo, Yan Zhao, Hongkai Zhang, Xu Yan, Hui Liu, Hailiang Li, Ihab R. Kamel, Jinrong Qu

**Affiliations:** 1 Department of Radiology, the Affiliated Cancer Hospital of Zhengzhou University & Henan Cancer Hospital, 127 Dongming Road, Zhengzhou, 450008 Henan China; 2 Advanced Application team, GE Healthcare, 1 Hua Tuo Road, Zhangjiang Hi-tech park, Pudong, Shanghai, 201203 China; 3 Department of Thoracic Surgery, the Affiliated Cancer Hospital of Zhengzhou University & Henan Cancer Hospital, 127 Dongming Road, Zhengzhou, 450008 Henan China; 4 National Cancer Center/Cancer Hospital, Chinese Academy of Medical Sciences and Peking Union Medical College, Beijing, 100021 China; 5 NEA MR Collaboration, Siemens Ltd, 278, Zhouzhu Road, Pudong New Area, Shanghai, 201318 China; 6 Department of Radiology, Johns Hopkins University School of Medicine, Baltimore, MD 21205-2196 USA

**Keywords:** Magnetic resonance imaging, Esophageal Cancer, Treatment outcome, Chemotherapy, Neoadjuvant therapy

## Abstract

**Background:**

To compare the value of two dynamic contrast-enhanced Magnetic Resonance Images (DCE-MRI) reconstruction approaches, namely golden-angle radial sparse parallel (GRASP) and view-sharing with golden-angle radial profile (VS-GR) reconstruction, and evaluate their values in assessing response to neoadjuvant chemotherapy (nCT) in patients with esophageal cancer (EC).

**Methods:**

EC patients receiving nCT before surgery were enrolled prospectively. DCE-MRI scanning was performed after nCT and within 1 week before surgery. Tumor Regression Grade (TRG) was used for chemotherapy response evaluation, and patients were stratified into a responsive group (TRG1 + 2) and a non-responsive group (TRG3 + 4 + 5). Wilcoxon test was utilized for comparing GRASP and VS-GR reconstruction, Kruskal-Wallis and Mann-Whitney test was performed for each parameter to assess response, and Spearman test was performed for analyzing correlation between parameters and TRGs, as well as responder and non-responder. The receiver operating characteristic (ROC) was utilized for each significant parameter to assess its accuracy between responders and non-responders.

**Results:**

Among the 64 patients included in this cohort (52 male, 12 female; average age of 59.1 ± 7.9 years), 4 patients showed TRG1, 4 patients were TRG2, 7 patients were TRG3, 11 patients were TRG4, and 38 patients were TRG5. They were stratified into 8 responders and 56 non-responders.

A total of 15 parameters were calculated from each tumor. With VS-GR, 10/15 parameters significantly correlated with TRG and response groups. Of these, only AUCmax showed moderate correlation with TRG, 7 showed low correlation and 2 showed negligible correlation with TRG. 8 showed low correlation and 2 showed negligible correlation with response groups. With GRASP, 13/15 parameters significantly correlated with TRG and response groups. Of these, 10 showed low correlation and 3 showed negligible correlation with TRG. 11 showed low correlation and 2 showed negligible correlation with TRG. Seven parameters (AUC^*^ > 0.70, *P* < 0.05) showed good performance in response groups.

**Conclusions:**

In patients with esophageal cancer on neoadjuvant chemotherapy, several parameters can differentiate responders from non-responders, using both GRASP and VS-GR techniques. GRASP may be able to better differentiate these two groups compared to VS-GR.

Trial registration for this prospective study: ChiCTR, ChiCTR-DOD-14005308. Registered 2 October 2014.

## Background

Esophageal cancer (EC) has become the eighth most common cancer, and the incidence rate is rising rapidly worldwide [1]. Squamous cell carcinoma (SCC) is the main pathological type of EC in China, and is a high-grade malignancy with rapid progression, poor response and high recurrence rate [2, 3]. Moreover, SCC is associated with limited quality of life after surgery, poor prognosis [4] and a high incidence of postoperative morbidity and mortality [5–7]. Ando et al. reported that nCT before resection is still the main treatment for stages II and III SCC [7, 8]. If local tumor is controlled, nCT followed by surgical procedures is an optimum treatment strategy, which can improve overall survival for patients with SCC [8]. Predicting response to nCT accurately helps clinicians to provide the best treatment approach such as modification of nCT, or termination of nCT to initiate surgical resection [1, 9].

18 F-fluorodeoxyglucose positron emission tomography (18 F-FDG-PET) shows to be a promising technique for predicting therapeutic response, but standardizing protocols and the time of scanning is required [10]. Dynamic contrast-enhanced Magnetic Resonance Images (DCE-MRI) have the ability to predict an early response in EC following 3 weeks of concurrent chemoradiotherapy in limited cases [11, 12]. However, it is still challenging to non-invasively predict response to nCT. Recently, golden-angle radial sparse parallel (GRASP) MRI has gained interest, and has been applied to imaging of the liver, rectal cancer and renal cell carcinoma [13–16]. GRASP is capable of reconstructing the acquired data at very high temporal resolution using only a small number of radial spokes for every temporal frame. This enables high-resolution free-breathing perfusion imaging with higher in-plane spatial resolution and thinner partitions. This results in near-isotropic resolution, compared with the current view-sharing with golden-angle radial profile (VS-GR) reconstruction, without the current imaging constraints of breath-holding techniques [13].

The aim of this study was to compare DCE-MRI with GRASP reconstruction to DCE-MRI with VS-GR reconstruction in assessing response to nCT in patients with EC and to identify DCE-MRI parameters that can differentiate responders from non-responders.

## Methods

**Fig. 1:**
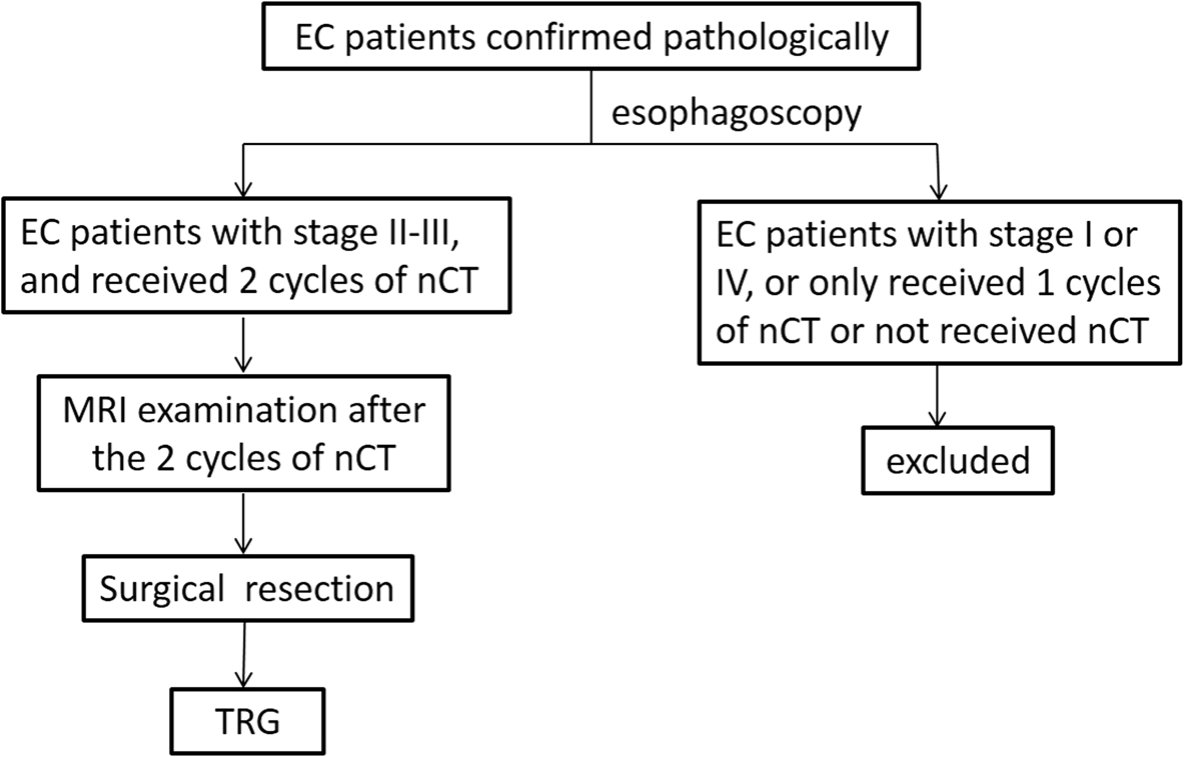
Flow chart illustrates patient selection process for study cohort

This prospective study was approved by the Ethics Committee of Henan Cancer Hospital (No.20140303), and written informed consent was obtained from all participants. Those patients who received nCT followed by surgical resection were enrolled. DCE-MRI was performed within 1 week before surgery. All studies were performed between September 2015 and March 2017. The inclusion criteria were following [17]: 1) Patients were confirmed with stage II-III EC by esophagoscopy pathologically [18, 19], 2) 2 cycles of nCT before surgery were performed, 3) Imaging and clinical response evaluation were performed at 2 weeks after completing all the treatment. (Fig. 1).

### DCE-MRI scanning methods

DCE-MRI examination was performed on a 3 T MR scanner (MAGNETOM Skyra, Siemens Healthcare) with dynamic contrast-enhanced Radial VIBE free breathing, and an 18-element body matrix coil and an inbuilt 32-element spine matrix coil were used. Radial VIBE sequence parameters were following: TR: 3.98 ms TE: 1.91 ms, flip angle: 12°, acquisition matrix: 300 × 300, FOV: 300 mm × 300 mm × 146 mm, slice thickness: 3 mm, reconstructed image voxel size: 1.0 × 1.0 × 3.0 mm^3^, radial views: 1659, scanning time: 309 s. A total of 68 period images were collected, and each period included 72 images. 10-15 mL Gadopentetate Dimeglumine Injection (0.2 ml/kg of body weight, Omniscan, GE Healthcare) was injected at a rate of 2.5 mL/s, followed by equal volume of normal saline solution to flush the tube at 20 s after the beginning of scanning by a MR-compatible automated high-pressure injector (Spectris Solaris EP, Medrad) [17].

### Histopathology response

Pathologic response was assessed as 5 grades according to Tumor Regression Grade (TRG) [20]: TRG 1 (complete regression) showed absence of residual cancer and fibrosis extending through the different layers of the esophageal wall; TRG 2 was characterized by the presence of rare residual cancer cells scattered through bands of fibrosis; TRG 3 was characterized by an increase in the number of residual cancer cells, but fibrosis still predominated; TRG 4 showed residual cancer outgrowing fibrosis; and TRG 5 was characterized by absence of regressive changes. They were stratified into a responsive group (TRG1 + 2) and a non-responsive group (TRG3 + 4 + 5).

### Image processing and data analysis

The radial views (1659 of stack-of-stars views acquired from DCE-MRI) were input into online reconstruction pipeline of view sharing reconstruction and regrouped into 2 sub-frames (sub-frame-1: T0-T61 with a temporal resolution of 2.4 s, sub-frame 2 from T62-T68 with temporal resolution of 21.7 s). A home setup of GRASP reconstruction processing pipeline (https://mrirecon.github.io/bart/) post processed on a Yarra server (https://yarra.rocks) were used for GRASPs offline, with the same data but using a temporal resolution of 4.5 s (Table 1).

**Table 1:** Details of reconstruction setting for radial VIBE with golden angle stack-of-stars sampling scheme

	View-Sharing	GRASP
number of acquired views	1659	
FOV	300 mm × 300 mm × 146 mm	
spatial resolution	1.0 × 1.0 × 3.0 mm^3^	
temporal resolution	2.4 s/21.7 s	4.5 s
number of dynamic volumes	68	68
Reconstruction mode	Online	Offline
Reconstruction time	N/A	62 minutes on a CPU server

Note: The temporal resolution of VS-GR means the starting time interval between two phases, however, 90% of the prior phase was overlapped with this phase. So, although the temporal resolution of VS-GR seems very short, actually it is longer

**Fig. 2:**
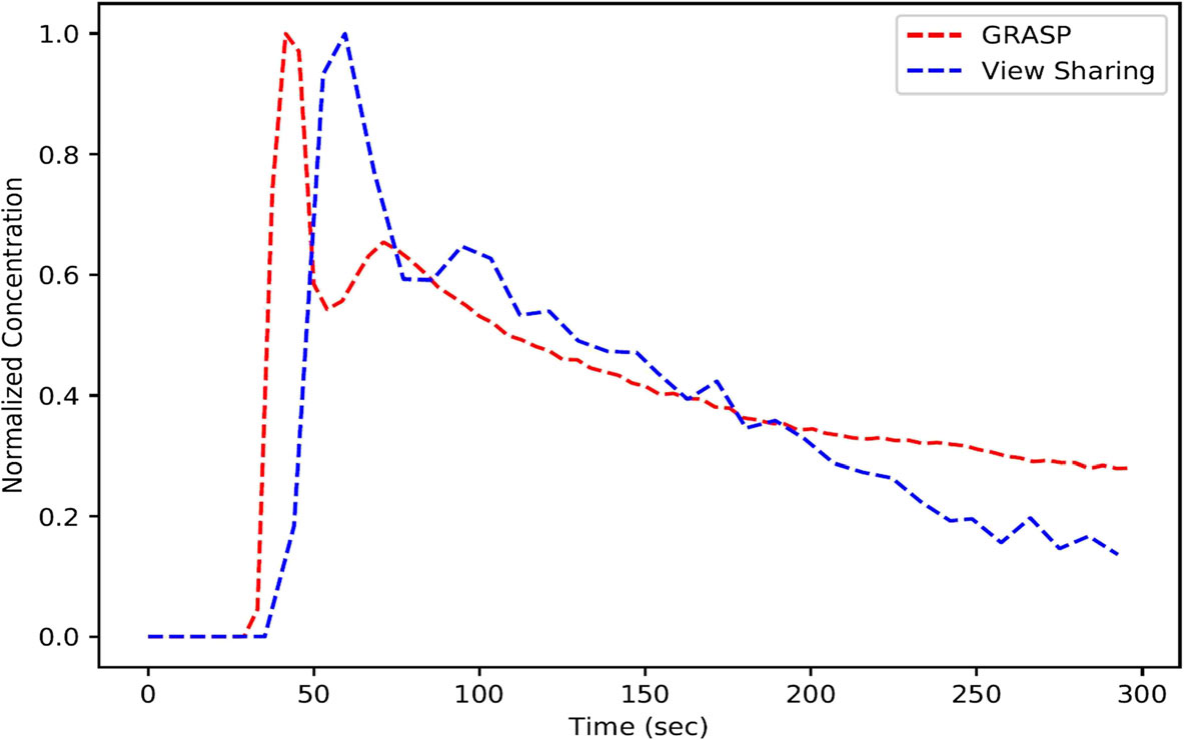
Arterial contrast concentration curve from GRASP (red) and view-sharing (blue) reconstruction using the same dynamic acquisition. GRASP’s AIF is closer to the true AIF with steeper slope and sharp peak than view-sharing

The images reconstructed by two different approaches, namely GRASP and VS-GR, were processed by Omni-Kinetics software (GE Medical, China) to segment the tumor and generate pharmacokinetic parameters respectively. The thoracic aorta was selected to obtain the arterial input function (AIF), since the esophageal artery is not easy to identify. Figure 2 shows the AIFs derived from GRASP and VS-GR reconstructions from the same contrast-enhanced study.

Two radiologists with more than 10 years experiences in thorax radiology segmented the 3D- regions of interest (ROI) manually. The radiologists were blinded to clinical data, and were asked to include the entire tumor on each slice post-nCT, except areas of necrotic degeneration or cystic and normal blood vessels. The pharmacokinetic parameters were generated by using Tofts model.

### Statistical analysis

SPSS Statistics version 22 (IBM Corp., Armonk, NY, USA) were used to perform statistical analysis in this study. Interobserver reproducibility of pharmacokinetic parameters was assessed by inter-class correlation coefficients (ICCs). An ICC > 0.75 was considered good agreement. The Wilcoxon test of was used to compare the various parameters between VS-GR and GRASP reconstruction, and Kruskal-Wallis test for DCE-MRI parameters with VS-GR or GRASP reconstruction among the TRG1–5 groups (*P* < 0.05). Mann-Whitney test was for analyzing the differences between responder and non-responder groups. Spearman test was performed for correlation analysis between DCE-MRI parameters and TRGs, or response groups. Spearman’s correlation coefficients were assessed as follows: a correlation coefficient of 0.90–1.00 is considered very high; 0.70–0.89, high; 0.50–0.69, moderate; 0.30–0.49, low; and 0–0.29, negligible [21]. The receiver operating characteristic (ROC) was adopted to assess the value of each parameter in predicting response (AUC^*^>0.50, *P*<0.05).

## Results

Among the total of 64 patients (52 male, 12 female, average age of 59.1 ± 7.9 years), 59 patients had SCC, 2 patients had adenocarcinoma and 3 patients had adenosquamous carcinoma. According to pathologic response, 4 patients showed TRG1, 4 patients were TRG2, 7 patients were TRG3, 11 patients were TRG4, and 38 patients were TRG5. They were stratified into 8 responders and 56 non-responders (Table 2).

**Table 2:** Patients’ demographic information and TRG

Study population	
Gender	
Male	52
Female	12
Age, years	59.1 ± 7.9
Clinical T-stage	
T1	2
T2	15
T3	42
T4	5
Clinical N-stage	
No	32
N1	15
N2	15
N3	2
Type	
SCC	59
AC	2
ASC	3
Tumor Regression Grade	
1	4
2	4
3	7
4	11
5	38

ICCs showed the excellence of 15 pharmacokinetic parameters from the two reconstructions as assessed by the two radiologists, and the kappa value was 0.918.

### Comparison of DCE-MRI parameters with VS-GR and GRASP reconstruction groups

GRASP showed a better AIF curve with steeper slope and sharper peak compared to VS-GR (Fig. 2). A total of 15 pharmacokinetic parameters were extracted from each tumor. 14 of these showed statistically significant difference for both VS-GR and GRASP reconstruction across the TRG groups. Only plasma volume fraction (Vp) max did not show a significant difference (*P* = 0.628).

### Comparison among TRG1–5 for DCE-MRI parameters with VS-GR and GRASP reconstruction

14/15 DCE-MRI parameters both with VS-GR and with GRASP reconstruction showed significant inter-groups difference by TRG 1–5 (*P* < 0.05), except for Ve max which showed not significant inter-groups difference by TRG 1–5 (Table 3).

**Table 3:** Differences among TRG1–5 for DCE-MRI parameters with VS-GR and GRASP reconstruction

parameters	VS-GR reconstruction							GRASP reconstruction						
	TRG1	TRG2	TRG3	TRG4	TRG5	χ^2^	*P* value	TRG1	TRG2	TRG3	TRG4	TRG5	χ^2^	*P* value
Ktrans max	0.000 (0.000,0.099)	1.075 (0.630,1.643)	0.713 (0.553,1.405)	2.477 (1.800,5.000)	2.396 (1.357,3.420)	20.101	<0.001	0.000 (0.000,0.086)	0.159 (0.100,0.304)	0.170 (0.117,0.207)	0.324 (0.198,0.905)	0.310 (0.200,0.424)	15.533	0.004
Ktrans mean	0.000 (0.000,0.037)	0.277 (0.105,0.343)	0.189 (0.094,0.369)	0.239 (0.200,0.549)	0.315 (0.166,0.405)	12.368	0.015	0.000 (0.000,0.027)	0.055 (0.030,0.106)	0.044 (0.025,0.068)	0.060 (0.045,0.126)	0.074 (0.051,0.095)	13.432	0.009
Ktrans 75%	0.000 (0.000,0.052)	0.359 (0.123,0.467)	0.307 (0.123,0.491)	0.372 (0.271,0.572)	0.422 (0.200,0.562)	12.313	0.015	0.000 (0.000,0.034)	0.082 (0.043,0.129)	0.056 (0.033,0.082)	0.074 (0.060,0.160)	0.091 (0.065,0.122)	12.531	0.014
Kep max	0.000 (0.000,0.200)	3.131 (2.127,5.898)	2.208 (1.657,3.024)	4.506 (2.729,7.424)	4.868 (2.823,6.578)	16.684	0.002	0.000 (0.000,0.489)	0.810 (0.543,1.410)	0.929 (0.580,1.121)	1.543 (1.010,1.985)	1.390 (0.854,1.877)	14.455	0.006
Kep mean	0.000 (0.000,0.002)	0.723 (0.388,0.824)	0.215 (0.113,0.493)	0.349 (0.252,1.185)	0.537 (0.361,0.844)	15.088	0.005	0.000 (0.000,0.123)	0.294 (0.167,0.558)	0.212 (0.167,0.455)	0.272 (0.213,0.434)	0.331 (0.227,0.432)	10.358	0.035
Kep 75%	0.000 (0.000,0.005)	1082 (0.833,1.568)	0.361 (0.010,0.799)	0.539 (0.327,1.571)	0.897 (0.545,1.361)	15.254	0.004	0.000 (0.000,0.160)	0.472 (0.262,0.664)	0.277 (0.228,0.532)	0.358 (0.274,0.552)	0.451 (0.282,0.540)	11.033	0.026
Ve max	1.000 (0.510,1.000)	1.000 (1.000,1.000)	1.000 (1.000,1.000)	1.000 (1.000,1.000)	1.000 (1.000,1.000)	7.180	0.127	1.000 (1.000,1.000)	0.824 (0.376,1.000)	1.000 (0.417,1.000)	1.000 (1.000,1.000)	1.000 (0.826,1.000)	3.447	0.486
Ve mean	0.000 (0.000,0.002)	O.349 (0.111,0.438)	0.369 (0.139,0.434)	0.331 (0.256,0.386)	0.317 (0.203,0.359)	11.552	0.021	0.000 (0.000,0.200)	0.196 (0.150,0.207)	0.189 (0.159,0.275)	0.230 (0.199,0.303)	0.230 (0.199,0.287)	9.809	0.044
Ve 75%	0.000 (0.000,0.0007)	0.387 (0.064,0.555)	0.505 (0.001,0.604)	0.503 (0.373,0.570)	0.468 (0.303,0.508)	11.478	0.022	0.000 (0.000,0.214)	0.220 (0.175,0.250)	0.219 (0.180,0.305)	0.257 (0.163,0.311)	0.256 (0.219,0.328)	8.518	0.074
Vp max	0.000 (0.000,0.007)	0.067 (0.033,0.115)	0.058 (0.041,0.101)	0.244 (0.056,0.630)	0.143 (0.073,0.249)	14.198	0.007	0.000 (0.000,0.034)	0.066 (0.050,0.104)	0.082 (0.045,0.137)	0.205 (0.103,0.335)	0.134 (0.090,0.236)	16.581	0.002
Vp mean	0.000 (0.000,0.0001)	0.004 (0.001,0.007)	0.001 (0.001,0.007)	0.003 (0.000,0.009)	0.002 (0.000,0.005)	9.772	0.044	0.000 (0.000,0.001)	0.009 (0.003,0.015)	0.003 (0.001,0.027)	0.019 (0.006,0.063)	0.014 (0.009,0.016)	17.041	0.002
Vp 75%	0.000 (0.000,0.0007)	0.001 (0.001,0.004)	0.001 (0.001,0.006)	0.001 (0.001,0.001)	0.001 (0.001,0.001)	9.602	0.048	0.000 (0.000,0008)	0.011 (0.003,0.022)	0.001 (0.001,0.041)	0.033 (0.007,0.080)	0.023 (0.014,0.038)	17.523	0.002
AUC max	0.000 (0.000,0.316)	0.071 (0.059,0.089)	0.083 (0.070,0.089)	0.101 (0.090,0.119)	0.121 (0.089,0.147)	21.365	<0.001	0.000 (0.000,1.619)	1.738 (0.974,2.350)	3.344 (2.259,6.248)	4.561 (2.326,6.424)	3.364 (2.516,4.512)	16.454	0.002
AUC mean	0.000 (0.000,0.012)	0.036 (0.018,0.045)	0.043 (0.026,0.045)	0.369 (0.032,0.041)	0.040 (0.029,0.047)	11.445	0.022	0.000 (0.000,0.793)	0.940 (0.562,1.469)	1.473 (1.036,1.617)	1.688 (1.035,2.375)	1.538 (1.190,1.875)	12.605	0.013
AUC 75%	0.000 (0.000,0.015)	0.045, (0.025,0.056)	0.052 (0.034,0.054)	0.047 (0.041,0.056)	0.053 (0.038,0.061)	11.980	0.018	0.000 (0.000,0.965)	1.180 (0.734,1.661)	1.642 (1.232,1.981)	2.041 (1.234,2.748)	1.854 (1.417,2.149)	12.994	0.011

Note. —Data are median (P25, P75)

### Comparison between responder and non-responder groups for DCE-MRI parameters with VS-GR/ GRASP reconstruction

Ten parameters with VS-GR reconstruction showed significant differences between responders and non-responders, which including volume transfer constant (Ktrans) max, Ktrans mean, Ktrans 75%, intravasation rate contrast (Kep) max, extravascular extracellular volume fraction (Ve) mean, Ve 75%, Vp max, the initial area-under-the- concentration versus time curve (AUC) max, AUC mean, AUC 75%. 13 parameters with GRASP reconstruction showed significant differences between responders and non-responders, which including Ktrans max, Ktrans mean, Ktrans 75%, Kep max, Kep mean, Ve mean, Ve 75%, Vp max, Vp mean, Vp 75%, AUC max, AUC mean, AUC 75% (Table 4).

**Table 4:** Differences between responder and non-responder groups for DCE-MRI parameters with VS-GR/GRASP reconstruction

parameters	VS-GR reconstruction				GRASP reconstruction			
	responder	non-responder	U value	*P* value	responder	non-responder	U value	*P* value
Ktrans max	0.314 (0.000,1.097)	2.303 (1.172,3.564)	51.0	<0.001	0.101 (0.000,0.189)	0.304 (0.196,0.415)	69.0	0.002
Ktrans mean	0.055 (0.000,0.298)	0.299 (0.157,0.387)	92.0	0.007	0.031 (0.000,0.067)	0.065 (0.046,0.094)	104.0	0.015
Ktrans 75%	0.069 (0.000,0.395)	0.404 (0.200,0.545)	92.0	0.007	0.043 (0.000,0.097)	0.082 (0.061,0.121)	115.0	0.027
Kep max	1.088 (0.000,3.309)	4.272 (2.307,6.527)	90.0	0.007	0.559 (0.000,0.934)	1.342 (0.861,1.875)	75.0	0.002
Kep mean	0.149 (0.000,0.751)	0.506 (0.251,0.823)	140.0	0.088	0.160 (0.000,0.340)	0.318 (0.212,0.432)	117.0	0.030
Kep 75%	0.384 (0.000,1.095)	0.772 (0.363,1.334)	154.0	0.155	0.215 (0.000,0.509)	0.429 (0.268,0.536)	137.0	0.077
Ve max	1.000 (1.000,1.000)	1.000 (1.000,1.000)	199.5	0.099	1.000 (0.736,1.000)	1.000 (0.875,1.000)	223.0	0.979
Ve mean	0.030 (0.000,0.388)	0.323 (0.201,0.370)	124.0	0.042	0.163 (0.000,0.207)	0.227 (0.189,0.276)	93.0	0.008
Ve 75%	0.006 (0.000,0.471)	0.475 (0.297,0.521)	112.0	0.023	0.187 (0.000,0.250)	0.250 (0.209,0.311)	104.0	0.015
Vp max	0.017 (0.000,0.068)	0.140 (0.056,0.316)	71.0	0.002	0.046 (0.000,0.070)	0.133 (0.085, 0.233)	63.0	0.001
Vp mean	0.0004 (0.0000,0.0048)	0.002 (0.001,0.006)	135.0	0.071	0.001 (0.000,0.009)	0.014 (0.008,0.030)	67.0	0.001
Vp 75%	0.001 (0.001,0.001)	0.001 (0.001,0.001)	142.0	0.096	0.001 (0.000,0.012)	0.023 (0.008,0.042)	65.0	0.001
AUC max	0.050 (0.000,0.076)	0.108 (0.085,0.137)	40.0	<0.001	1.062 (0.000,2.220)	3.424 (2.489,4.832)	31.0	<0.001
AUC mean	0.016 (0.000,0.040)	0.039 (0.030,0.047)	90.0	0.007	0.569 (0.000,1.236)	1.515 (1.188,1.901)	60.0	0.001
AUC 75%	0.020 (0.000,0.049)	0.052 (0.052,0.060)	86.0	0.005	0.753 (0.000,1.497)	1.854 (1.403,2.203)	55.0	0.001

Note. —Data are median (P25, P75)

### Correlation between parameters with VS-GR/GRASP reconstruction and TRG/response

With VS-GR, 10/15 parameters significantly correlated with TRG and response groups. Of these, only AUCmax showed moderate correlation with TRG, 7 showed low correlation and 2 showed negligible correlation with TRG. 8 showed low correlation and 2 showed negligible correlation with response groups. With GRASP, 13/15 parameters significantly correlated with TRG and response groups. Of these, 10 showed low correlation and 3 showed negligible correlation with TRG. 11 showed low correlation and 2 showed negligible correlation with TRGs (Table 5).

**Table 5:** DCE-MRI parameters with VS-GR/GRASP stratified according to TRGs and response

Parameters	TRG1–5		responder and non-responder	
	VS-GR	GRASP	VS-GR	GRASP
	r*(*P*)	r* (*P*)	r* (*P*)	r* (*P*)
Ktrans max	0.409 (0.001)	0.343 (0.006)	0.443(< 0.001)	0.396 (0.001)
Ktrans mean	0.305 (0.014)	0.320 (0.010)	0.338 (0.006)	0.307 (0.014)
Ktrans 75%	0.318 (0.011)	0.282 (0.024)	0.338 (0.006)	0.279 (0.026)
Kep max	0.379 (0.002)	0.323 (0.009)	0.343 (0.006)	0.381 (0.002)
Kep mean	0.314 (0.012)	0.255 (0.042)	0.215 (0.088)	0.274 (0.029)
Kep 75%	0.283 (0.023)	0.238 (0.058)	0.179 (0.157)	0.223 (0.077)
Ve max	0.097 (0.446)	−0.035 (0.784)	0.208 (0.099)	−0.003 (0.979)
Ve mean	0.125 (0.324)	0.318 (0.010)	0.256 (0.041)	0.335 (0.007)
Ve 75%	0.151 (0.234)	0.330 (0.008)	0.286 (0.022)	0.307 (0.014)
Vp max	0.312 (0.012)	0.333 (0.007)	0.391 (0.001)	0.412 (0.001)
Vp mean	0.158 (0.213)	0.371 (0.003)	0.228 (0.070)	0.402 (0.001)
Vp 75%	0.115 (0.366)	0.370 (0.003)	0.210 (0.096)	0.407 (0.001)
AUC max	0.524(< 0.001)	0.253 (0.044)	0.471(< 0.001)	0.494(< 0.001)
AUC mean	0.294 (0.018)	0.306 (0.014)	0.343 (0.006)	0.419 (0.001)
AUC 75%	0.314 (0.012)	0.307 (0.014)	0.353 (0.004)	0.432(< 0.001)

Note.—r* is the Spearman correlation coefficient obtained from the nonparametric Spearman correlation test

### Diagnostic performance of DCE-MRI parameters with VS-GR/ GRASP reconstruction between responder and non-responder groups

Seven parameters with VS-GR/GRASP reconstruction showed good or excellent diagnostic performance between responders and non-responders, which including Ktrans max, Ktrans mean, Kep max, Vp max, AUC max, AUC mean, AUC 75%. In general, the seven variables had similar diagnostic performance in the two reconstructions. Among the seven variables, AUC max showed excellent performance in response groups (AUC^*^>0.90, *P*<0.05) (Table 6).

**Table 6:** Diagnostic performance of DCE-MRI parameters with VS-GR/GRASP according to response groups

Parameters	Sensitivity (%)		Speciticity (%)		AUC^*^		*P*
	VS-GR	GRASP	VS-GR	GRASP	VS-GR	GRASP	
Ktrans max	87.5	75.0	76.8	85.7	0.886	0.846	0.464
Ktrans mean	62.5	75.0	98.2	83.9	0.795	0.768	0.634
Ktrans 75%	62.5	75.0	98.2	78.6	0.795	0.743	0.256
Kep max	50.0	75.0	100.0	83.9	0.799	0.833	0.708
Kep mean	50.0	75.0	100.0	76.8	0.687	0.739	0.566
Kep 75%	50.0	62.5	100.0	87.5	0.656	0.694	0.658
Ve max	12.5	87.5	100.0	3.6	0.555	0.502	0.604
Ve mean	62.5	87.5	98.2	64.3	0.723	0.792	0.672
Ve 75%	75.0	75.0	78.6	67.9	0.750	0.768	0.905
Vp max	87.5	87.5	71.4	78.6	0.842	0.859	0.785
Vp mean	62.5	62.5	85.7	96.4	0.699	0.850	0.221
Vp 75%	62.5	62.5	82.1	98.2	0.683	0.855	0.211
AUC max	87.5	100.0	82.1	80.4	0.911	0.931	0.580
AUC mean	62.5	62.5	96.4	98.2	0.799	0.866	0.249
AUC 75%	62.5	62.5	96.4	98.2	0.808	0.877	0.280

## Discussion

This study demonstrated that GRASP reconstruction may affect the results of DCE-MRI, DCE-MRI with VS-GR and GRASP reconstruction could assess tumor response, and pharmacokinetic parameters with GRASP and VS-GR reconstruction may help stratify responders from non-responders in patients with EC treated by nCT. In this study, 10 post-nCT pharmacokinetic parameters with VS-GR reconstruction and 13 parameters with GRASP reconstruction showed statistically significant differences between responders and non-responders. Moreover, GRASP reconstruction provided more parameters than VS-GR reconstruction. However, seven parameters with VS-GR/GRASP reconstruction showed good or excellent diagnostic performance between responders and non-responders and no significant difference in diagnostic performance between VS-GR and GRASP reconstructions.

Most DCE-MRI studies only analyzed parts of parameters, such as Ktrans mean, kep mean, Ve mean, and AUC, and showed DCE-MRI could assess the response to therapy [22]. In the current study, we tried to analyze more parameters acquired from DCE-MRI, and 15 parameters were analyzed.

It was reported that DCE-MRI with GRASP reconstruction could provide near-isotropic resolution and higher in-plane spatial resolution [13]. Contribution to the VS-GR images with a 2.1 s apparent temporal resolution is from a ~ 21 s time footprint acquisition, while GRASP is reconstructed from a 4.5 s time footprint, higher temporal resolution normally leads to an improved AIF, which is used for more accurate pharmacokinetics parameters calculation [23]. Compared to conventional VS-GR DCE-MRI, this could result in better acquisition of pharmacokinetic parameters potentially which has been reported in hepatocellular carcinoma, renal cell carcinoma and rectal cancer [13] [16] [15]. VS-GR DCE-MRI had been used in EC [12], however, GRASP reconstruction has not been reported to be compared with VS-GR reconstruction in EC. The AIF plays an important role for the pharmacokinetic models in determining the quantitative measurements of physiological parameters, where small differences in AIF may lead to large differences in quantitative maps and higher temporal resolution gives smaller differences.

More parameters with GRASP showed significant correlation with TRGs and response groups than those with VS-GR reconstruction. Both 10/15 parameters with VS-GR reconstruction showed significant correlation with TRGs and response groups, and both 13/15 parameters with GRASP reconstruction showed significant correlation with TRGs and response groups. It may be the effect of GRASP reconstruction, providing higher time resolution and more information.

It is critical to detecting residual cancer post-nT. Fortunately, some pharmacokinetic parameters between TGRs showed significant differences in this study. The information of whole tumor, rather than a single axial level, was assessed in our study, which theoretically provides a more comprehensive representation of tumor information than that provided by a single-level analysis.

FDG-PET have been used for neoadjuvant treatment response assessment in EC [24], and the FDG-PET response after neoadjuvant treatment could predict the pathological response and seems to be related to survival [25–27]. However, Van Rossum et al. showed that accuracy of imaging is insufficient in predicting pathologic response [28], and the prognostic value of FDG-PET response after chemoradiotherapy has not been definitively established [29, 30].

There were several limitations in this study. First, one critical step in quantifying DCE MRI parameters is to sample AIF from a major artery. However, to sample AIF in esophageal images can be challenging, because of its small size. Li et al. showed automatically sampling AIF by utilizing temporal and spatial features in a multistep interleaved manner, that highly resembled those manually sampled ones in lower extremity arteries [31]. Second, limited sample size, the number of TRG1 in particular, may lead to bias. Finally, GRASP reconstruction required offline reconstruction, more computing ability and more time for reconstruction.

## Conclusions

Several pharmacokinetic parameters of DCE-MRI reconstructed by GRASP and VS-GR show significant differences between TRGs and response groups and thus can be used to non-invasively predict tumor response. GRASP reconstruction provided more parameters than VS-GR reconstruction, which maybe showed additionally significant merit, and larger sample size study need to assess it furtherly.

## Data Availability

The datasets used and/or analyzed during the current study are available from the corresponding author on reasonable request.

## Abbreviations

AIF: Arterial input function
AUC: Area under the ROC curve
AUC: the initial area-under-the- concentration versus time curve
DCE-MRI: Dynamic contrast-enhanced Magnetic resonance imaging
EC: Esophageal cancer
GRASP: Golden-angle radial sparse parallel
Kep: Rate contrast
Ktrans: Volume transfer constant
nCT: Neoadjuvant chemotherapy
ROC: Receiver operating characteristic
ROI: Regions of interest
TRG: Tumor Regression Grade
Ve: Extravascular extracellular volume fraction
Vp: Plasma volume fraction
VS-GR: View-sharing with golden-angle radial profile
